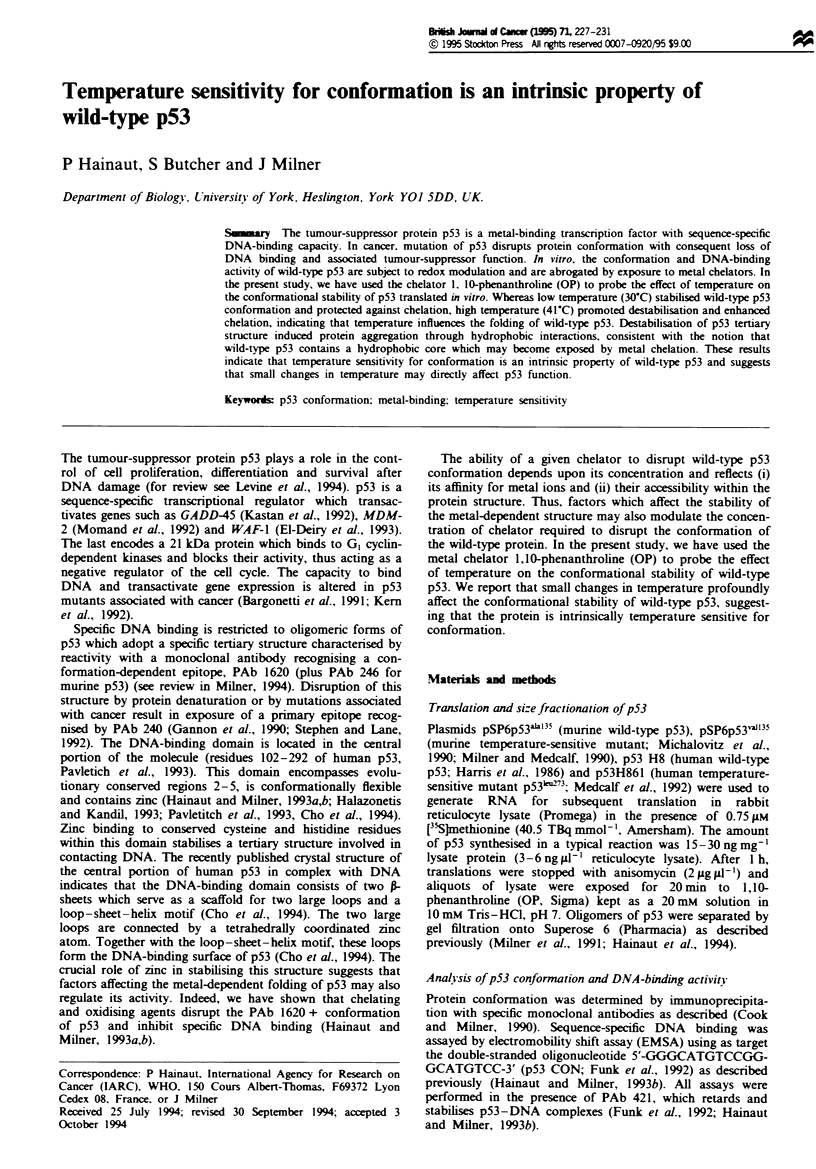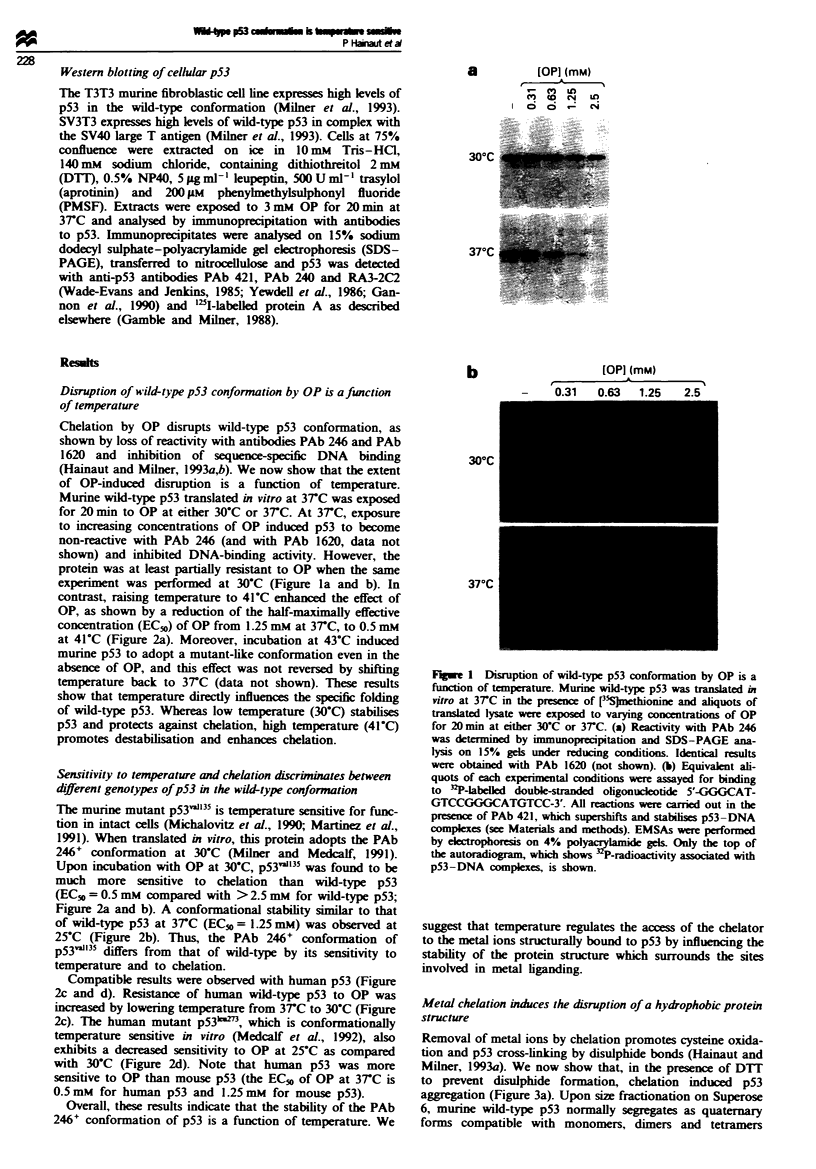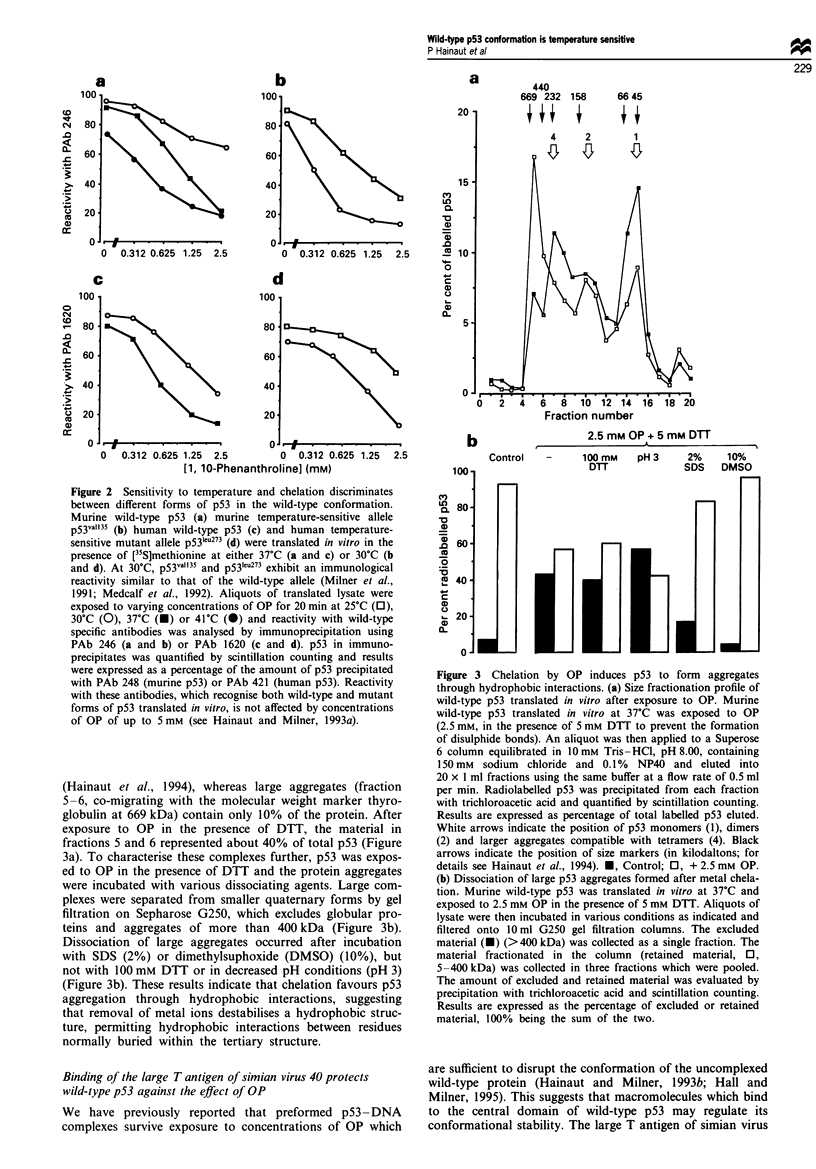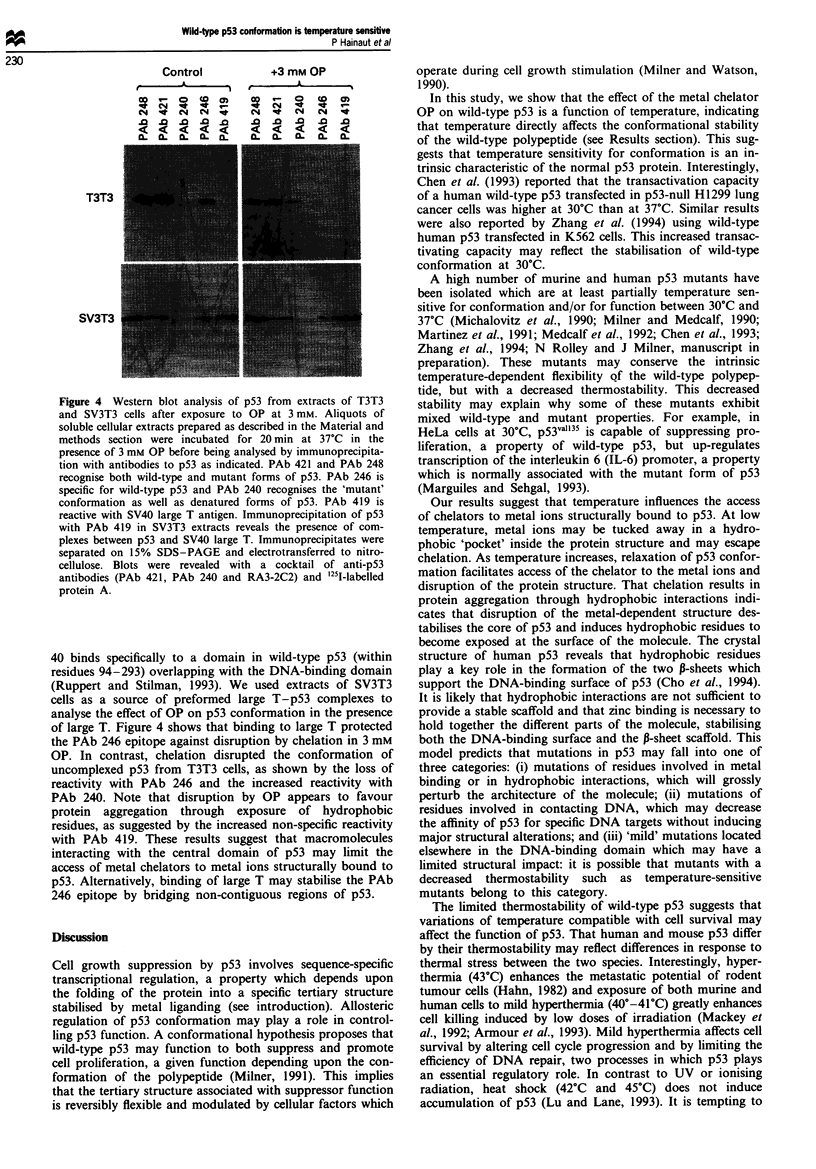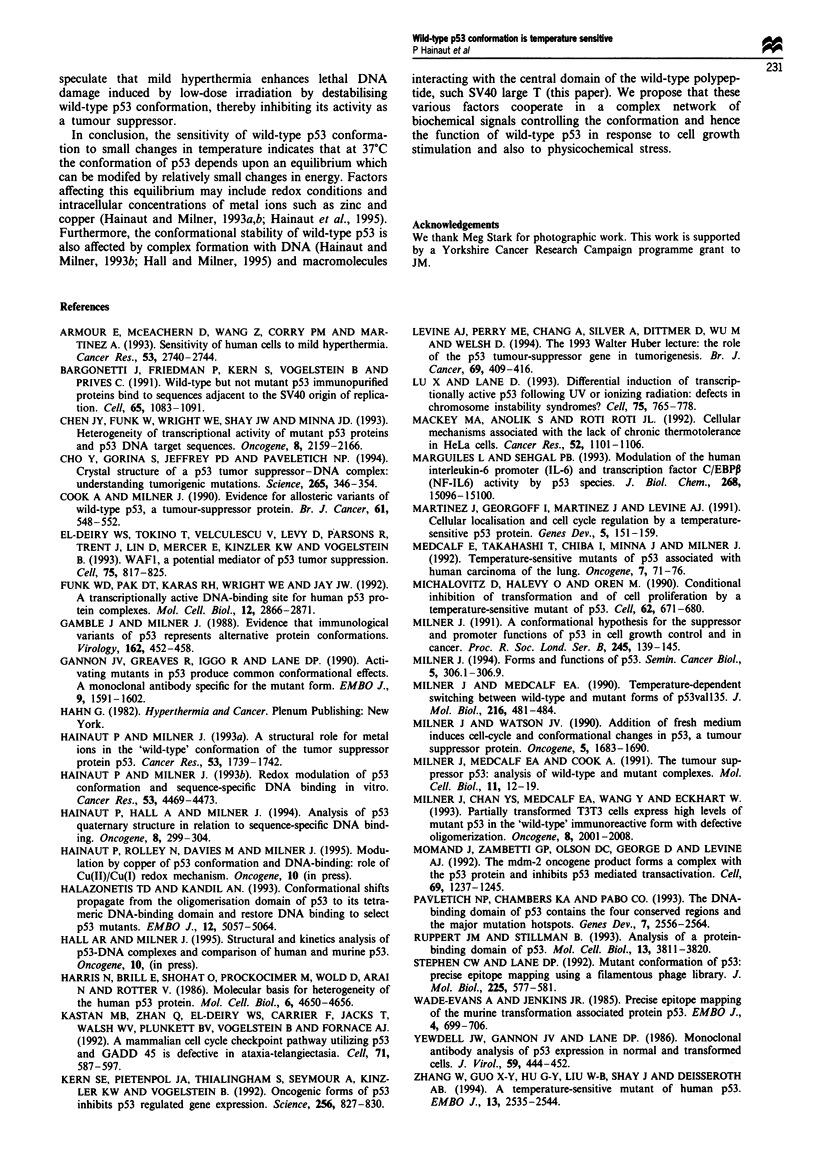# Temperature sensitivity for conformation is an intrinsic property of wild-type p53.

**DOI:** 10.1038/bjc.1995.48

**Published:** 1995-02

**Authors:** P. Hainaut, S. Butcher, J. Milner

**Affiliations:** Department of Biology, University of York, Heslington, U.K.

## Abstract

**Images:**


British Journal of Cancer (1995) 71, 227-231

© 1995 Stockton Press All rights reserved 0007-0920/95 $9.00

### Temperature sensitivity for conformation is an intrinsic property of wild-type p53

P Hainaut, S Butcher and J Milner

*Department of Biology, University of York, Heslington, York YO1 5DD, UK.*

**Summary** The tumour-suppressor protein p53 is a metal-binding transcription factor with sequence-specific DNA-binding capacity. In cancer, mutation of p53 disrupts protein conformation with consequent loss of DNA binding and associated tumour-suppressor function. *In vitro*, the conformation and DNA-binding activity of wild-type p53 are subject to redox modulation and are abrogated by exposure to metal chelators. In the present study, we have used the chelator 1, 10-phenanthroline (OP) to probe the effect of temperature on the conformational stability of p53 translated *in vitro*. Whereas low temperature (30°C) stabilised wild-type p53 conformation and protected against chelation, high temperature (41°C) promoted destabilisation and enhanced chelation, indicating that temperature influences the folding of wild-type p53. Destabilisation of p53 tertiary structure induced protein aggregation through hydrophobic interactions, consistent with the notion that wild-type p53 contains a hydrophobic core which may become exposed by metal chelation. These results indicate that temperature sensitivity for conformation is an intrinsic property of wild-type p53 and suggests that small changes in temperature may directly affect p53 function.

**Keywords:** p53 conformation; metal-binding; temperature sensitivity

The tumour-suppressor protein p53 plays a role in the control of cell proliferation, differentiation and survival after DNA damage (for review see Levine *et al.*, 1994). p53 is a sequence-specific transcriptional regulator which transactivates genes such as *GADD-45* (Kastan *et al.*, 1992), *MDM-2* (Momand *et al.*, 1992) and *WAF-1* (El-Deiry *et al.*, 1993). The last encodes a 21 kDa protein which binds to $G_1$ cyclin-dependent kinases and blocks their activity, thus acting as a negative regulator of the cell cycle. The capacity to bind DNA and transactivate gene expression is altered in p53 mutants associated with cancer (Bargonetti *et al.*, 1991; Kern *et al.*, 1992).

Specific DNA binding is restricted to oligomeric forms of p53 which adopt a specific tertiary structure characterised by reactivity with a monoclonal antibody recognising a conformation-dependent epitope, PAb 1620 (plus PAb 246 for murine p53) (see review in Milner, 1994). Disruption of this structure by protein denaturation or by mutations associated with cancer result in exposure of a primary epitope recognised by PAb 240 (Gannon *et al.*, 1990; Stephen and Lane, 1992). The DNA-binding domain is located in the central portion of the molecule (residues 102-292 of human p53, Pavletich *et al.*, 1993). This domain encompasses evolutionary conserved regions 2-5, is conformationally flexible and contains zinc (Hainaut and Milner, 1993*a*,*b*; Halazonetis and Kandil, 1993; Pavletitch *et al.*, 1993, Cho *et al.*, 1994). Zinc binding to conserved cysteine and histidine residues within this domain stabilises a tertiary structure involved in contacting DNA. The recently published crystal structure of the central portion of human p53 in complex with DNA indicates that the DNA-binding domain consists of two β-sheets which serve as a scaffold for two large loops and a loop-sheet-helix motif (Cho *et al.*, 1994). The two large loops are connected by a tetrahedrally coordinated zinc atom. Together with the loop-sheet-helix motif, these loops form the DNA-binding surface of p53 (Cho *et al.*, 1994). The crucial role of zinc in stabilising this structure suggests that factors affecting the metal-dependent folding of p53 may also regulate its activity. Indeed, we have shown that chelating and oxidising agents disrupt the PAb 1620+ conformation of p53 and inhibit specific DNA binding (Hainaut and Milner, 1993*a*,*b*).

Correspondence: P Hainaut, International Agency for Research on Cancer (IARC), WHO, 150 Cours Albert-Thomas, F69372 Lyon Cedex 08, France, or J Milner
Received 25 July 1994; revised 30 September 1994; accepted 3 October 1994

The ability of a given chelator to disrupt wild-type p53 conformation depends upon its concentration and reflects (i) its affinity for metal ions and (ii) their accessibility within the protein structure. Thus, factors which affect the stability of the metal-dependent structure may also modulate the concentration of chelator required to disrupt the conformation of the wild-type protein. In the present study, we have used the metal chelator 1,10-phenanthroline (OP) to probe the effect of temperature on the conformational stability of wild-type p53. We report that small changes in temperature profoundly affect the conformational stability of wild-type p53, suggesting that the protein is intrinsically temperature sensitive for conformation.

### Materials and methods

### *Translation and size fractionation of p53*

Plasmids pSP6p53$^{ala135}$ (murine wild-type p53), pSP6p53$^{val135}$ (murine temperature-sensitive mutant; Michalovitz *et al.*, 1990; Milner and Medcalf, 1990), p53 H8 (human wild-type p53; Harris *et al.*, 1986) and p53H861 (human temperature-sensitive mutant p53$^{leu273}$; Medcalf *et al.*, 1992) were used to generate RNA for subsequent translation in rabbit reticulocyte lysate (Promega) in the presence of 0.75 μM [$^{35}$S]methionine (40.5 TBq mmol$^{-1}$, Amersham). The amount of p53 synthesised in a typical reaction was 15-30 ng mg$^{-1}$ lysate protein (3-6 ng μl$^{-1}$ reticulocyte lysate). After 1 h, translations were stopped with anisomycin (2 μg μl$^{-1}$) and aliquots of lysate were exposed for 20 min to 1,10-phenanthroline (OP, Sigma) kept as a 20 mM solution in 10 mM Tris-HCl, pH 7. Oligomers of p53 were separated by gel filtration onto Superose 6 (Pharmacia) as described previously (Milner *et al.*, 1991; Hainaut *et al.*, 1994).

### *Analysis of p53 conformation and DNA-binding activity*

Protein conformation was determined by immunoprecipitation with specific monoclonal antibodies as described (Cook and Milner, 1990). Sequence-specific DNA binding was assayed by electromobility shift assay (EMSA) using as target the double-stranded oligonucleotide 5'-GGGCATGTCCGG-GCATGTCC-3' (p53 CON; Funk *et al.*, 1992) as described previously (Hainaut and Milner, 1993*b*). All assays were performed in the presence of PAb 421, which retards and stabilises p53-DNA complexes (Funk *et al.*, 1992; Hainaut and Milner, 1993*b*).

*Western blotting of cellular p53*

The T3T3 murine fibroblastic cell line expresses high levels of p53 in the wild-type conformation (Milner *et al*., 1993). SV3T3 expresses high levels of wild-type p53 in complex with the SV40 large T antigen (Milner *et al*., 1993). Cells at 75% confluence were extracted on ice in 10 mM Tris-HCl, 140 mM sodium chloride, containing dithiothreitol 2 mM (DTT), 0.5% NP40, 5 μg $ml^{-1}$ leupeptin, 500 U $ml^{-1}$ trasylol (aprotinin) and 200 μM phenylmethylsulphonyl fluoride (PMSF). Extracts were exposed to 3 mM OP for 20 min at 37°C and analysed by immunoprecipitation with antibodies to p53. Immunoprecipitates were analysed on 15% sodium dodecyl sulphate-polyacrylamide gel electrophoresis (SDS-PAGE), transferred to nitrocellulose and p53 was detected with anti-p53 antibodies PAb 421, PAb 240 and RA3-2C2 (Wade-Evans and Jenkins, 1985; Yewdell *et al*., 1986; Gannon *et al*., 1990) and $^{125}$I-labelled protein A as described elsewhere (Gamble and Milner, 1988).

### Results

*Disruption of wild-type p53 conformation by OP is a function of temperature*

Chelation by OP disrupts wild-type p53 conformation, as shown by loss of reactivity with antibodies PAb 246 and PAb 1620 and inhibition of sequence-specific DNA binding (Hainaut and Milner, 1993*a*,*b*). We now show that the extent of OP-induced disruption is a function of temperature. Murine wild-type p53 translated *in vitro* at 37°C was exposed for 20 min to OP at either 30°C or 37°C. At 37°C, exposure to increasing concentrations of OP induced p53 to become non-reactive with PAb 246 (and with PAb 1620, data not shown) and inhibited DNA-binding activity. However, the protein was at least partially resistant to OP when the same experiment was performed at 30°C (Figure 1a and b). In contrast, raising temperature to 41°C enhanced the effect of OP, as shown by a reduction of the half-maximally effective concentration ($EC_{50}$) of OP from 1.25 mM at 37°C, to 0.5 mM at 41°C (Figure 2a). Moreover, incubation at 43°C induced murine p53 to adopt a mutant-like conformation even in the absence of OP, and this effect was not reversed by shifting temperature back to 37°C (data not shown). These results show that temperature directly influences the specific folding of wild-type p53. Whereas low temperature (30°C) stabilises p53 and protects against chelation, high temperature (41°C) promotes destabilisation and enhances chelation.

*Sensitivity to temperature and chelation discriminates between different genotypes of p53 in the wild-type conformation*

The murine mutant $p53^{val135}$ is temperature sensitive for function in intact cells (Michalovitz *et al*., 1990; Martinez *et al*., 1991). When translated *in vitro*, this protein adopts the PAb $246^+$ conformation at 30°C (Milner and Medcalf, 1991). Upon incubation with OP at 30°C, $p53^{val135}$ was found to be much more sensitive to chelation than wild-type p53 ($EC_{50} = 0.5$ mM compared with $>2.5$ mM for wild-type p53; Figure 2a and b). A conformational stability similar to that of wild-type p53 at 37°C ($EC_{50} = 1.25$ mM) was observed at 25°C (Figure 2b). Thus, the PAb $246^+$ conformation of $p53^{val135}$ differs from that of wild-type by its sensitivity to temperature and to chelation.

Compatible results were observed with human p53 (Figure 2c and d). Resistance of human wild-type p53 to OP was increased by lowering temperature from 37°C to 30°C (Figure 2c). The human mutant $p53^{leu273}$, which is conformationally temperature sensitive *in vitro* (Medcalf *et al*., 1992), also exhibits a decreased sensitivity to OP at 25°C as compared with 30°C (Figure 2d). Note that human p53 was more sensitive to OP than mouse p53 (the $EC_{50}$ of OP at 37°C is 0.5 mM for human p53 and 1.25 mM for mouse p53).

Overall, these results indicate that the stability of the PAb $246^+$ conformation of p53 is a function of temperature. We suggest that temperature regulates the access of the chelator to the metal ions structurally bound to p53 by influencing the stability of the protein structure which surrounds the sites involved in metal liganding.

**Figure 1** Disruption of wild-type p53 conformation by OP is a function of temperature. Murine wild-type p53 was translated *in vitro* at 37°C in the presence of [$^{35}$S]methionine and aliquots of translated lysate were exposed to varying concentrations of OP for 20 min at either 30°C or 37°C. (**a**) Reactivity with PAb 246 was determined by immunoprecipitation and SDS-PAGE analysis on 15% gels under reducing conditions. Identical results were obtained with PAb 1620 (not shown). (**b**) Equivalent aliquots of each experimental conditions were assayed for binding to $^{32}$P-labelled double-stranded oligonucleotide 5′-GGGCAT-GTCCGGGCATGTCC-3′. All reactions were carried out in the presence of PAb 421, which supershifts and stabilises p53-DNA complexes (see Materials and methods). EMSAs were performed by electrophoresis on 4% polyacrylamide gels. Only the top of the autoradiogram, which shows $^{32}$P-radioactivity associated with p53-DNA complexes, is shown.

*Metal chelation induces the disruption of a hydrophobic protein structure*

Removal of metal ions by chelation promotes cysteine oxidation and p53 cross-linking by disulphide bonds (Hainaut and Milner, 1993*a*). We now show that, in the presence of DTT to prevent disulphide formation, chelation induced p53 aggregation (Figure 3a). Upon size fractionation on Superose 6, murine wild-type p53 normally segregates as quaternary forms compatible with monomers, dimers and tetramers

**Figure 2** Sensitivity to temperature and chelation discriminates between different forms of p53 in the wild-type conformation. Murine wild-type p53 (**a**) murine temperature-sensitive allele $p53^{val135}$ (**b**) human wild-type p53 (**c**) and human temperature-sensitive mutant allele $p53^{leu273}$ (**d**) were translated *in vitro* in the presence of [$^{35}$S]methionine at either 37°C (**a** and **c**) or 30°C (**b** and **d**). At 30°C, $p53^{val135}$ and $p53^{leu273}$ exhibit an immunological reactivity similar to that of the wild-type allele (Milner *et al.*, 1991; Medcalf *et al.*, 1992). Aliquots of translated lysate were exposed to varying concentrations of OP for 20 min at 25°C (□), 30°C (○), 37°C (■) or 41°C (●) and reactivity with wild-type specific antibodies was analysed by immunoprecipitation using PAb 246 (**a** and **b**) or PAb 1620 (**c** and **d**). p53 in immunoprecipitates was quantified by scintillation counting and results were expressed as a percentage of the amount of p53 precipitated with PAb 248 (murine p53) or PAb 421 (human p53). Reactivity with these antibodies, which recognise both wild-type and mutant forms of p53 translated *in vitro*, is not affected by concentrations of OP of up to 5 mM (see Hainaut and Milner, 1993*a*).

(Hainaut *et al.*, 1994), whereas large aggregates (fraction 5–6, co-migrating with the molecular weight marker thyroglobulin at 669 kDa) contain only 10% of the protein. After exposure to OP in the presence of DTT, the material in fractions 5 and 6 represented about 40% of total p53 (Figure 3a). To characterise these complexes further, p53 was exposed to OP in the presence of DTT and the protein aggregates were incubated with various dissociating agents. Large complexes were separated from smaller quaternary forms by gel filtration on Sepharose G250, which excludes globular proteins and aggregates of more than 400 kDa (Figure 3b). Dissociation of large aggregates occurred after incubation with SDS (2%) or dimethylsuphoxide (DMSO) (10%), but not with 100 mM DTT or in decreased pH conditions (pH 3) (Figure 3b). These results indicate that chelation favours p53 aggregation through hydrophobic interactions, suggesting that removal of metal ions destabilises a hydrophobic structure, permitting hydrophobic interactions between residues normally buried within the tertiary structure.

*Binding of the large T antigen of simian virus 40 protects wild-type p53 against the effect of OP*

We have previously reported that preformed p53–DNA complexes survive exposure to concentrations of OP which

**Figure 3** Chelation by OP induces p53 to form aggregates through hydrophobic interactions. (**a**) Size fractionation profile of wild-type p53 translated *in vitro* after exposure to OP. Murine wild-type p53 translated *in vitro* at 37°C was exposed to OP (2.5 mM, in the presence of 5 mM DTT to prevent the formation of disulphide bonds). An aliquot was then applied to a Superose 6 column equilibrated in 10 mM Tris–HCl, pH 8.00, containing 150 mM sodium chloride and 0.1% NP40 and eluted into 20 × 1 ml fractions using the same buffer at a flow rate of 0.5 ml per min. Radiolabelled p53 was precipitated from each fraction with trichloroacetic acid and quantified by scintillation counting. Results are expressed as percentage of total labelled p53 eluted. White arrows indicate the position of p53 monomers (1), dimers (2) and larger aggregates compatible with tetramers (4). Black arrows indicate the position of size markers (in kilodaltons; for details see Hainaut *et al.*, 1994). ■, Control; □, + 2.5 mM OP. (**b**) Dissociation of large p53 aggregates formed after metal chelation. Murine wild-type p53 was translated *in vitro* at 37°C and exposed to 2.5 mM OP in the presence of 5 mM DTT. Aliquots of lysate were then incubated in various conditions as indicated and filtered onto 10 ml G250 gel filtration columns. The excluded material (■) (>400 kDa) was collected as a single fraction. The material fractionated in the column (retained material, □, 5–400 kDa) was collected in three fractions which were pooled. The amount of excluded and retained material was evaluated by precipitation with trichloroacetic acid and scintillation counting. Results are expressed as the percentage of excluded or retained material, 100% being the sum of the two.

are sufficient to disrupt the conformation of the uncomplexed wild-type protein (Hainaut and Milner, 1993*b*; Hall and Milner, 1995). This suggests that macromolecules which bind to the central domain of wild-type p53 may regulate its conformational stability. The large T antigen of simian virus

**Figure 4** Western blot analysis of p53 from extracts of T3T3 and SV3T3 cells after exposure to OP at 3 mM. Aliquots of soluble cellular extracts prepared as described in the Material and methods section were incubated for 20 min at 37°C in the presence of 3 mM OP before being analysed by immunoprecipitation with antibodies to p53 as indicated. PAb 421 and PAb 248 recognise both wild-type and mutant forms of p53. PAb 246 is specific for wild-type p53 and PAb 240 recognises the 'mutant' conformation as well as denatured forms of p53. PAb 419 is reactive with SV40 large T antigen. Immunoprecipitation of p53 with PAb 419 in SV3T3 extracts reveals the presence of complexes between p53 and SV40 large T. Immunoprecipitates were separated on 15% SDS-PAGE and electrotransferred to nitrocellulose. Blots were revealed with a cocktail of anti-p53 antibodies (PAb 421, PAb 240 and RA3-2C2) and $^{125}$I-labelled protein A.

40 binds specifically to a domain in wild-type p53 (within residues 94–293) overlapping with the DNA-binding domain (Ruppert and Stilman, 1993). We used extracts of SV3T3 cells as a source of preformed large T–p53 complexes to analyse the effect of OP on p53 conformation in the presence of large T. Figure 4 shows that binding to large T protected the PAb 246 epitope against disruption by chelation in 3 mM OP. In contrast, chelation disrupted the conformation of uncomplexed p53 from T3T3 cells, as shown by the loss of reactivity with PAb 246 and the increased reactivity with PAb 240. Note that disruption by OP appears to favour protein aggregation through exposure of hydrophobic residues, as suggested by the increased non-specific reactivity with PAb 419. These results suggest that macromolecules interacting with the central domain of p53 may limit the access of metal chelators to metal ions structurally bound to p53. Alternatively, binding of large T may stabilise the PAb 246 epitope by bridging non-contiguous regions of p53.

### Discussion

Cell growth suppression by p53 involves sequence-specific transcriptional regulation, a property which depends upon the folding of the protein into a specific tertiary structure stabilised by metal liganding (see introduction). Allosteric regulation of p53 conformation may play a role in controlling p53 function. A conformational hypothesis proposes that wild-type p53 may function to both suppress and promote cell proliferation, a given function depending upon the conformation of the polypeptide (Milner, 1991). This implies that the tertiary structure associated with suppressor function is reversibly flexible and modulated by cellular factors which operate during cell growth stimulation (Milner and Watson, 1990).

In this study, we show that the effect of the metal chelator OP on wild-type p53 is a function of temperature, indicating that temperature directly affects the conformational stability of the wild-type polypeptide (see Results section). This suggests that temperature sensitivity for conformation is an intrinsic characteristic of the normal p53 protein. Interestingly, Chen *et al.* (1993) reported that the transactivation capacity of a human wild-type p53 transfected in p53-null H1299 lung cancer cells was higher at 30°C than at 37°C. Similar results were also reported by Zhang *et al.* (1994) using wild-type human p53 transfected in K562 cells. This increased transactivating capacity may reflect the stabilisation of wild-type conformation at 30°C.

A high number of murine and human p53 mutants have been isolated which are at least partially temperature sensitive for conformation and/or for function between 30°C and 37°C (Michalovitz *et al.*, 1990; Milner and Medcalf, 1990; Martinez *et al.*, 1991; Medcalf *et al.*, 1992; Chen *et al.*, 1993; Zhang *et al.*, 1994; N Rolley and J Milner, manuscript in preparation). These mutants may conserve the intrinsic temperature-dependent flexibility of the wild-type polypeptide, but with a decreased thermostability. This decreased stability may explain why some of these mutants exhibit mixed wild-type and mutant properties. For example, in HeLa cells at 30°C, p53$^{val135}$ is capable of suppressing proliferation, a property of wild-type p53, but up-regulates transcription of the interleukin 6 (IL-6) promoter, a property which is normally associated with the mutant form of p53 (Marguiles and Sehgal, 1993).

Our results suggest that temperature influences the access of chelators to metal ions structurally bound to p53. At low temperature, metal ions may be tucked away in a hydrophobic 'pocket' inside the protein structure and may escape chelation. As temperature increases, relaxation of p53 conformation facilitates access of the chelator to the metal ions and disruption of the protein structure. That chelation results in protein aggregation through hydrophobic interactions indicates that disruption of the metal-dependent structure destabilises the core of p53 and induces hydrophobic residues to become exposed at the surface of the molecule. The crystal structure of human p53 reveals that hydrophobic residues play a key role in the formation of the two β-sheets which support the DNA-binding surface of p53 (Cho *et al.*, 1994). It is likely that hydrophobic interactions are not sufficient to provide a stable scaffold and that zinc binding is necessary to hold together the different parts of the molecule, stabilising both the DNA-binding surface and the β-sheet scaffold. This model predicts that mutations in p53 may fall into one of three categories: (i) mutations of residues involved in metal binding or in hydrophobic interactions, which will grossly perturb the architecture of the molecule; (ii) mutations of residues involved in contacting DNA, which may decrease the affinity of p53 for specific DNA targets without inducing major structural alterations; and (iii) 'mild' mutations located elsewhere in the DNA-binding domain which may have a limited structural impact: it is possible that mutants with a decreased thermostability such as temperature-sensitive mutants belong to this category.

The limited thermostability of wild-type p53 suggests that variations of temperature compatible with cell survival may affect the function of p53. That human and mouse p53 differ by their thermostability may reflect differences in response to thermal stress between the two species. Interestingly, hyperthermia (43°C) enhances the metastatic potential of rodent tumour cells (Hahn, 1982) and exposure of both murine and human cells to mild hyperthermia (40°–41°C) greatly enhances cell killing induced by low doses of irradiation (Mackey *et al.*, 1992; Armour *et al.*, 1993). Mild hyperthermia affects cell survival by altering cell cycle progression and by limiting the efficiency of DNA repair, two processes in which p53 plays an essential regulatory role. In contrast to UV or ionising radiation, heat shock (42°C and 45°C) does not induce accumulation of p53 (Lu and Lane, 1993). It is tempting to speculate that mild hyperthermia enhances lethal DNA damage induced by low-dose irradiation by destabilising wild-type p53 conformation, thereby inhibiting its activity as a tumour suppressor.

In conclusion, the sensitivity of wild-type p53 conformation to small changes in temperature indicates that at 37°C the conformation of p53 depends upon an equilibrium which can be modifed by relatively small changes in energy. Factors affecting this equilibrium may include redox conditions and intracellular concentrations of metal ions such as zinc and copper (Hainaut and Milner, 1993*a*,*b*; Hainaut *et al.*, 1995). Furthermore, the conformational stability of wild-type p53 is also affected by complex formation with DNA (Hainaut and Milner, 1993*b*; Hall and Milner, 1995) and macromolecules interacting with the central domain of the wild-type polypeptide, such SV40 large T (this paper). We propose that these various factors cooperate in a complex network of biochemical signals controlling the conformation and hence the function of wild-type p53 in response to cell growth stimulation and also to physicochemical stress.

**Acknowledgements**
We thank Meg Stark for photographic work. This work is supported by a Yorkshire Cancer Research Campaign programme grant to JM.

### References

ARMOUR E, MCEACHERN D, WANG Z, CORRY PM AND MARTINEZ A. (1993). Sensitivity of human cells to mild hyperthermia. *Cancer Res.*, **53**, 2740–2744.

BARGONETTI J, FRIEDMAN P, KERN S, VOGELSTEIN B AND PRIVES C. (1991). Wild-type but not mutant p53 immunopurified proteins bind to sequences adjacent to the SV40 origin of replication. *Cell*, **65**, 1083–1091.

CHEN JY, FUNK W, WRIGHT WE, SHAY JW AND MINNA JD. (1993). Heterogeneity of transcriptional activity of mutant p53 proteins and p53 DNA target sequences. *Oncogene*, **8**, 2159–2166.

CHO Y, GORINA S, JEFFREY PD AND PAVELETICH NP. (1994). Crystal structure of a p53 tumor suppressor–DNA complex: understanding tumorigenic mutations. *Science*, **265**, 346–354.

COOK A AND MILNER J. (1990). Evidence for allosteric variants of wild-type p53, a tumour-suppressor protein. *Br. J. Cancer*, **61**, 548–552.

EL-DEIRY WS, TOKINO T, VELCULESCU V, LEVY D, PARSONS R, TRENT J, LIN D, MERCER E, KINZLER KW AND VOGELSTEIN B. (1993). WAF1, a potential mediator of p53 tumor suppression. *Cell*, **75**, 817–825.

FUNK WD, PAK DT, KARAS RH, WRIGHT WE AND JAY JW. (1992). A transcriptionally active DNA-binding site for human p53 protein complexes. *Mol. Cell. Biol.*, **12**, 2866–2871.

GAMBLE J AND MILNER J. (1988). Evidence that immunological variants of p53 represents alternative protein conformations. *Virology*, **162**, 452–458.

GANNON JV, GREAVES R, IGGO R AND LANE DP. (1990). Activating mutants in p53 produce common conformational effects. A monoclonal antibody specific for the mutant form. *EMBO J.*, **9**, 1591–1602.

HAHN G. (1982). *Hyperthermia and Cancer*. Plenum Publishing: New York.

HAINAUT P AND MILNER J. (1993*a*). A structural role for metal ions in the 'wild-type' conformation of the tumor suppressor protein p53. *Cancer Res.*, **53**, 1739–1742.

HAINAUT P AND MILNER J. (1993*b*). Redox modulation of p53 conformation and sequence-specific DNA binding in vitro. *Cancer Res.*, **53**, 4469–4473.

HAINAUT P, HALL A AND MILNER J. (1994). Analysis of p53 quaternary structure in relation to sequence-specific DNA binding. *Oncogene*, **8**, 299–304.

HAINAUT P, ROLLEY N, DAVIES M AND MILNER J. (1995). Modulation by copper of p53 conformation and DNA-binding: role of Cu(II)/Cu(I) redox mechanism. *Oncogene*, **10** (in press).

HALAZONETIS TD AND KANDIL AN. (1993). Conformational shifts propagate from the oligomerisation domain of p53 to its tetrameric DNA-binding domain and restore DNA binding to select p53 mutants. *EMBO J.*, **12**, 5057–5064.

HALL AR AND MILNER J. (1995). Structural and kinetics analysis of p53-DNA complexes and comparison of human and murine p53. *Oncogene*, **10**, (in press).

HARRIS N, BRILL E, SHOHAT O, PROCKOCIMER M, WOLD D, ARAI N AND ROTTER V. (1986). Molecular basis for heterogeneity of the human p53 protein. *Mol. Cell. Biol.*, **6**, 4650–4656.

KASTAN MB, ZHAN Q, EL-DEIRY WS, CARRIER F, JACKS T, WALSH WV, PLUNKETT BV, VOGELSTEIN B AND FORNACE AJ. (1992). A mammalian cell cycle checkpoint pathway utilizing p53 and GADD 45 is defective in ataxia-telangiectasia. *Cell*, **71**, 587–597.

KERN SE, PIETENPOL JA, THIALINGHAM S, SEYMOUR A, KINZLER KW AND VOGELSTEIN B. (1992). Oncogenic forms of p53 inhibits p53 regulated gene expression. *Science*, **256**, 827–830.

LEVINE AJ, PERRY ME, CHANG A, SILVER A, DITTMER D, WU M AND WELSH D. (1994). The 1993 Walter Huber lecture: the role of the p53 tumour-suppressor gene in tumorigenesis. *Br. J. Cancer*, **69**, 409–416.

LU X AND LANE D. (1993). Differential induction of transcriptionally active p53 following UV or ionizing radiation: defects in chromosome instability syndromes? *Cell*, **75**, 765–778.

MACKEY MA, ANOLIK S AND ROTI ROTI JL. (1992). Cellular mechanisms associated with the lack of chronic thermotolerance in HeLa cells. *Cancer Res.*, **52**, 1101–1106.

MARGUILES L AND SEHGAL PB. (1993). Modulation of the human interleukin-6 promoter (IL-6) and transcription factor C/EBPβ (NF-IL6) activity by p53 species. *J. Biol. Chem.*, **268**, 15096–15100.

MARTINEZ J, GEORGOFF I, MARTINEZ J AND LEVINE AJ. (1991). Cellular localisation and cell cycle regulation by a temperature-sensitive p53 protein. *Genes Dev.*, **5**, 151–159.

MEDCALF E, TAKAHASHI T, CHIBA I, MINNA J AND MILNER J. (1992). Temperature-sensitive mutants of p53 associated with human carcinoma of the lung. *Oncogene*, **7**, 71–76.

MICHALOVITZ D, HALEVY O AND OREN M. (1990). Conditional inhibition of transformation and of cell proliferation by a temperature-sensitive mutant of p53. *Cell*, **62**, 671–680.

MILNER J. (1991). A conformational hypothesis for the suppressor and promoter functions of p53 in cell growth control and in cancer. *Proc. R. Soc. Lond. Ser. B*, **245**, 139–145.

MILNER J. (1994). Forms and functions of p53. *Semin. Cancer Biol.*, **5**, 306.1–306.9.

MILNER J AND MEDCALF EA. (1990). Temperature-dependent switching between wild-type and mutant forms of p53val135. *J. Mol. Biol.*, **216**, 481–484.

MILNER J AND WATSON JV. (1990). Addition of fresh medium induces cell-cycle and conformational changes in p53, a tumour suppressor protein. *Oncogene*, **5**, 1683–1690.

MILNER J, MEDCALF EA AND COOK A. (1991). The tumour suppressor p53: analysis of wild-type and mutant complexes. *Mol. Cell. Biol.*, **11**, 12–19.

MILNER J, CHAN YS, MEDCALF EA, WANG Y AND ECKHART W. (1993). Partially transformed T3T3 cells express high levels of mutant p53 in the 'wild-type' immunoreactive form with defective oligomerization. *Oncogene*, **8**, 2001–2008.

MOMAND J, ZAMBETTI GP, OLSON DC, GEORGE D AND LEVINE AJ. (1992). The mdm-2 oncogene product forms a complex with the p53 protein and inhibits p53 mediated transactivation. *Cell*, **69**, 1237–1245.

PAVLETICH NP, CHAMBERS KA AND PABO CO. (1993). The DNA-binding domain of p53 contains the four conserved regions and the major mutation hotspots. *Genes Dev.*, **7**, 2556–2564.

RUPPERT JM AND STILLMAN B. (1993). Analysis of a protein-binding domain of p53. *Mol. Cell. Biol.*, **13**, 3811–3820.

STEPHEN CW AND LANE DP. (1992). Mutant conformation of p53: precise epitope mapping using a filamentous phage library. *J. Mol. Biol.*, **225**, 577–581.

WADE-EVANS A AND JENKINS JR. (1985). Precise epitope mapping of the murine transformation associated protein p53. *EMBO J.*, **4**, 699–706.

YEWDELL JW, GANNON JV AND LANE DP. (1986). Monoclonal antibody analysis of p53 expression in normal and transformed cells. *J. Virol.*, **59**, 444–452.

ZHANG W, GUO X-Y, HU G-Y, LIU W-B, SHAY J AND DEISSEROTH AB. (1994). A temperature-sensitive mutant of human p53. *EMBO J.*, **13**, 2535–2544.